# Safety of User-Initiated Intensification of Insulin Delivery Using Cambridge Hybrid Closed-Loop Algorithm

**DOI:** 10.1177/19322968221141924

**Published:** 2022-12-08

**Authors:** Julia Ware, Malgorzata E. Wilinska, Yue Ruan, Janet M. Allen, Charlotte K. Boughton, Sara Hartnell, Lia Bally, Carine de Beaufort, Rachel E. J. Besser, Fiona M. Campbell, Katharine Draxlbauer, Daniela Elleri, Mark L. Evans, Elke Fröhlich-Reiterer, Atrayee Ghatak, Sabine E. Hofer, Thomas M. Kapellen, Lalantha Leelarathna, Julia K. Mader, Womba M. Mubita, Parth Narendran, Tina Poettler, Birgit Rami-Merhar, Martin Tauschmann, Tabitha Randell, Hood Thabit, Ajay Thankamony, Nicola Trevelyan, Roman Hovorka

**Affiliations:** 1 Metabolic Research Laboratories, Wellcome Trust-MRC Institute of Metabolic Science, University of Cambridge, Cambridge, UK; 2 Department of Paediatrics, University of Cambridge, Cambridge, UK; 3 Department of Diabetes and Endocrinology, Cambridge University Hospitals NHS Foundation Trust, Cambridge, UK; 4 Department of Diabetes, Endocrinology, Nutritional Medicine and Metabolism, Inselspital, Bern University Hospital, Bern, Switzerland; 5 Diabetes & Endocrine Care Clinique Pediatrique, Centre Hospitalier de Luxembourg, Luxembourg City, Luxembourg; 6 Department of Paediatric Endocrinology, UZ-VUB, Brussels, Belgium; 7 NIHR Oxford Biomedical Research Centre, Oxford University Hospitals NHS Foundation Trust, Oxford, UK; 8 Department of Paediatrics, University of Oxford, Oxford, UK; 9 Department of Paediatric Diabetes, Leeds Children’s Hospital, Leeds, UK; 10 University Hospitals Birmingham NHS Foundation Trust, Birmingham, UK; 11 Department of Diabetes, Royal Hospital for Sick Children, Edinburgh, UK; 12 Department of Pediatric and Adolescent Medicine, Medical University of Graz, Graz, Austria; 13 Department of Diabetes, Alder Hey Children’s NHS Foundation Trust, Liverpool, UK; 14 Department of Pediatrics I, Medical University of Innsbruck, Innsbruck, Austria; 15 Hospital for Children and Adolescents, Leipzig University, Leipzig, Germany; 16 Diabetes, Endocrinology and Metabolism Centre, Manchester University NHS Foundation Trust, Manchester Academic Health Science Centre, Manchester, UK; 17 Division of Diabetes, Endocrinology & Gastroenterology, Faculty of Biology, Medicine and Health, The University of Manchester, Manchester, UK; 18 Division of Endocrinology and Diabetology, Department of Internal Medicine, Medical University of Graz, Graz, Austria; 19 Institute of Immunology and Immunotherapy, University of Birmingham, Birmingham, UK; 20 Department of Paediatrics and Adolescent Medicine, Medical University of Vienna, Vienna, Austria; 21 Department of Paediatric Diabetes and Endocrinology, Nottingham Children’s Hospital, Nottingham University Hospitals NHS Trust, Nottingham, UK; 22 Department of Paediatric Endocrinology and Diabetes, Southampton Children’s Hospital, Southampton General Hospital, Southampton, UK

**Keywords:** artificial pancreas, automated insulin delivery, closed-loop, hypoglycemia, personalized medicine, type 1 diabetes

## Abstract

**Objective:**

Many hybrid closed-loop (HCL) systems struggle to manage unusually high glucose levels as experienced with intercurrent illness or pre-menstrually. Manual correction boluses may be needed, increasing hypoglycemia risk with overcorrection. The Cambridge HCL system includes a user-initiated algorithm intensification mode (“Boost”), activation of which increases automated insulin delivery by approximately 35%, while remaining glucose-responsive. In this analysis, we assessed the safety of “Boost” mode.

**Methods:**

We retrospectively analyzed data from closed-loop studies involving young children (1-7 years, *n* = 24), children and adolescents (10-17 years, *n* = 19), adults (≥24 years, *n* = 13), and older adults (≥60 years, *n* = 20) with type 1 diabetes. Outcomes were calculated per participant for days with ≥30 minutes of “Boost” use versus days with no “Boost” use. Participants with <10 “Boost” days were excluded. The main outcome was time spent in hypoglycemia <70 and <54 mg/dL.

**Results:**

Eight weeks of data for 76 participants were analyzed. There was no difference in time spent <70 and <54 mg/dL between “Boost” days and “non-Boost” days; mean difference: –0.10% (95% confidence interval [CI] –0.28 to 0.07; *P* = .249) time <70 mg/dL, and 0.03 (–0.04 to 0.09; *P* = .416) time < 54 mg/dL. Time in significant hyperglycemia >300 mg/dL was 1.39 percentage points (1.01 to 1.77; *P* < .001) higher on “Boost” days, with higher mean glucose and lower time in target range (*P* < .001).

**Conclusions:**

Use of an algorithm intensification mode in HCL therapy is safe across all age groups with type 1 diabetes. The higher time in hyperglycemia observed on “Boost” days suggests that users are more likely to use algorithm intensification on days with extreme hyperglycemic excursions.

## Introduction

Automated insulin delivery systems are becoming increasingly common in clinical practice as more such systems are becoming available.^1,2^ All currently available systems use the hybrid closed-loop (HCL) approach, where the closed-loop algorithm automatically adjusts insulin delivery based on real-time sensor glucose levels, but user-initiated prandial boluses are required for optimal outcomes. These systems have been shown to improve glycemic control and reduce time in hypoglycemia.^3-6^ There are however limitations. Systems may struggle to cope with unusually high glucose levels and/or unusually high insulin requirements, as experienced with intercurrent illness, in the pre-menstrual period, following high-fat meals or during pubertal or growth hormone surges. This is often due to inherent safety mitigations that do not allow algorithm-driven insulin delivery to exceed pre-specified amounts. Consequently, users need to administer manual correction boluses to bring glucose back into the target range, contributing to management burden and risk of hypoglycemia with overcorrection.^7^

Qualitative research shows that people with type 1 diabetes increasingly expect closed-loop systems to be able to cope with atypical scenarios.^8-10^ In response, some closed-loop systems, such as the CamAPS FX app, now include a personalizable user-initiated mode of operation, activation of which notifies the algorithm that insulin requirements are higher for a user-defined time period.^11^ This has the potential to improve usability, increase time in range, and reduce risk of hypoglycemia associated with standard manual correction doses. However, the safety of this specific user-initiated mode of operation that allows intensification of insulin delivery has not been evaluated. In the present analysis, we explore the relationship between user-initiated intensification of insulin delivery and time spent with glucose levels below the target range when using the Cambridge HCL algorithm, hypothesizing that use of “Boost” mode is not associated with an increase in time spent in hypoglycemia.

## Methods

### Study Population

We retrospectively analyzed data from four multicenter randomized clinical trials conducted in children, adolescents, adults, and older adults with type 1 diabetes, aged 1 to 80 years.^4,5,12,13^ All participants used the CamAPS FX HCL app for a minimum period of eight weeks in the unsupervised home setting. Study participants and parents/caregivers of participants signed informed consent; in line with local ethics committee recommendations, written assent was obtained from minors whenever possible. The studies were approved by independent research ethics committees and national regulatory authorities. Inclusion criteria for the studies included type 1 diabetes diagnosis (World Health Organization criteria) for a minimum of six months (young children) or 12 months (older adults), pump therapy for a minimum of three months, and a baseline glycated hemoglobin of <10% (all except adolescents).

### Closed-Loop System

All participants used the CamAPS FX closed-loop app (CamDiab Ltd, Cambridge, UK), running on an unlocked android smartphone and communicating via Bluetooth with the Dana Diabecare RS insulin pump (Sooil Development, Seoul, Korea) and Dexcom G6 (Dexcom, San Diego, CA, USA) continuous glucose monitor (CGM). In this HCL system, a model-predictive control algorithm incorporating adaptive learning automatically adjusts insulin delivery every 8 to 12 minutes to achieve a default nominal glucose target of 104.5 mg/dL. CamAPS FX offers personalizable, user-initiated modes of operation, including “Boost” and “Ease off” modes. Activating “Ease off” mode raises the personal glucose target and reduces algorithm-driven insulin delivery, and additionally stops any insulin delivery if glucose drops below 126 mg/dL. “Boost” mode increases algorithm-driven insulin delivery by approximately 35% for a user-defined period, while remaining glucose-responsive. “Boost” can be used in circumstances of unusually high glucose levels or increased insulin requirements, such as during the pre-menstrual period, for low-grade illness in the absence of significant ketones, to correct post-prandial hyperglycemia or during growth hormone pulses. Once target glucose is reached “Boost” mode will become inactive, irrespective of pre-programmed duration. Study participants were free to use “Boost” mode at any time during the study period; however, no data were collected on reasons for “Boost” use.

### Statistical Analysis

For each participant, the following outcomes were calculated per 24-hour segment starting at 00:00 and ending at 23:59 over the eight-week data collection period: time in “Boost” mode; percentage of time in closed-loop; percentage of time with CGM available; mean sensor glucose, standard deviation (SD) and coefficient of variation of sensor glucose; percentage of time with sensor glucose below 70 and 54 mg/dL; percentage of time with sensor glucose above 300 mg/dL; and daily total, basal, and bolus insulin dose. Participant days with <70% time in closed-loop were excluded from the analysis.

To explore the association between “Boost”-use and time in hypoglycemia, each participant day was categorized according to “Boost”-use: Either as “non-Boost day” (time with “Boost” active = 0 minutes) or “Boost day” (time with “Boost” active ≥ 30 minutes). Participant days with “Boost”-use between 1 and 29 minutes were excluded from the analysis. Mean glycemic metrics for all non-Boost days and all Boost days were then calculated per participant. Participants who had <10 non-Boost days or <10 Boost days were excluded from the analysis. Glycemic metrics on Boost versus non-Boost days were compared using a paired *t*-test, non-normally distributed data were winsorized.

Outcomes were calculated using GStat software, version 2.3 (University of Cambridge, UK), and statistical analyses were performed using SPSS, version 27 (IBM Software, Hampshire, UK). Measures are reported as mean ± SD for normally distributed or median (interquartile range [IQR]) for non-normally distributed data. *P* values < .05 were considered statistically significant.

## Results

Data from 143 participants aged between 1 and 80 years were analyzed over an eight-week period of HCL use in the home setting. Out of 7983 available days, 7378 days (92%) met the pre-specified inclusion criteria of at least 70% closed-loop use and Boost use of either 0 minutes or ≥ 30 minutes; 76 participants had ≥10 days of Boost use (median 23 days [IQR: 15-29] per participant), and were included in the final analysis.

Glycemic and closed-loop specific outcomes are shown in Table 1. The cohort included 24 children aged 1 to 7 years, 19 children and adolescents aged 10 to 17 years, 13 adults aged 24 to 55 years, and 20 older adults aged 60 to 80 years; 54% of the cohort were men, with an overall mean age of 29.4 ± 12.9 years. Closed-loop usage was high across all age groups at median 99%. The overall mean time with sensor glucose in the target range of 70 to 180 mg/dL was 75% ± 9%. The overall median time “Boost” was active per participant per day was 0.0% (IQR: 0.0-6.2; equivalent of 0.0-89.3 minutes). Time spent in hypoglycemia <70 mg/dL was low overall (2.6% [1.6-4.4]), and highest in very young children (5.2% [3.2-7.7]).

**Table 1.** Participant Characteristics and Outcomes by Study Cohort.

	Overall (n = 76)	Very young children^a^ (n = 24)	Children and adolescents^a^ (n = 19)	Adults^a^ (n = 13)	Older adults^a^ (n=20)
Age (yr)	29.4 ± 12.9	5.2 ± 1.4	12.4 ± 1.6	39.5 ± 9.8	68.2 ± 4.5
Sex—no. (%)					
Female	35 (46)	12 (50)	9 (47)	6 (46)	8 (40)
Male	41 (54)	12 (50)	10 (53)	7 (54)	12 (60)
Usage					
Time using CGM (%)	97 ± 4	95 ± 5	96 ± 3	97 ± 3	99 ± 2
Time in closed-loop (%)	99 (93-100)	99 (94-100)	98 (91-100)	99 (95-100)	99 (96-100)
Time in “Boost” (%)	0.0 (0.0-6.2)	0.0 (0.0-6.9)	0.0 (0.0-6.3)	0.0 (0.0-4.2)	0.0 (0.0-6.3)
Sensor glucose					
Time in range 70-180 mg/dL (%)	75 ± 9	74 ± 7	71 ± 11	74 ± 6	79 ± 9
Mean glucose (mg/dL)	145.4 ± 16.0	141.1 ± 13.8	153.2 ± 20.4	146.4 ± 9.1	142.9 ± 15.3
SD (mg/dL)	54.1 ± 12.2	55.3 ± 9.8	62.2 ± 15.5	54.7 ± 6.8	44.5 ± 6.8
Coefficient of variation (%)	37 ± 6	39 ± 5	40 ± 6	37 ± 3	31 ± 3
Time < 70 mg/dL (%)	2.6 (1.6-4.4)	5.2 (3.2-7.7)	2.4 (2.0-3.5)	3.2 (1.7-4.0)	1.5 (1.2-2.2)
Time < 54 mg/dL (%)	0.4 (0.2-1.0)	1.2 (0.5-1.6)	0.4 (0.2-0.7)	0.7 (0.3-0.8)	0.1 (0.1-0.3)
Time > 300 mg/dL (%)	1.0 (0.4-2.6)	1.3 (0.6-2.6)	4.0 (0.9-6.0)	1.4 (0.9-2.3)	0.5 (0.1-0.8)
Insulin delivery					
Total daily insulin dose (U/day)	42 ± 25	17 ± 5	65 ± 29	48 ± 14	45 ± 12
Total daily basal insulin dose (U/day)	24 ± 18	8 ± 3	40 ± 24	29 ± 10	26 ± 9
Total daily bolus insulin dose (U/day)	17 ± 8	9 ± 3	25 ± 9	19 ± 5	19 ± 6

Data are mean ± SD or median (IQR) unless otherwise indicated.

Abbreviations: CGM, continuous glucose monitor; IQR, interquartile range; SD, standard deviation.

a Very young children were aged 1 to 7 years, children and adolescents 10 to 17 years, adults 24 to 55 years, and older adults 65+ years.

**Figure 1.**
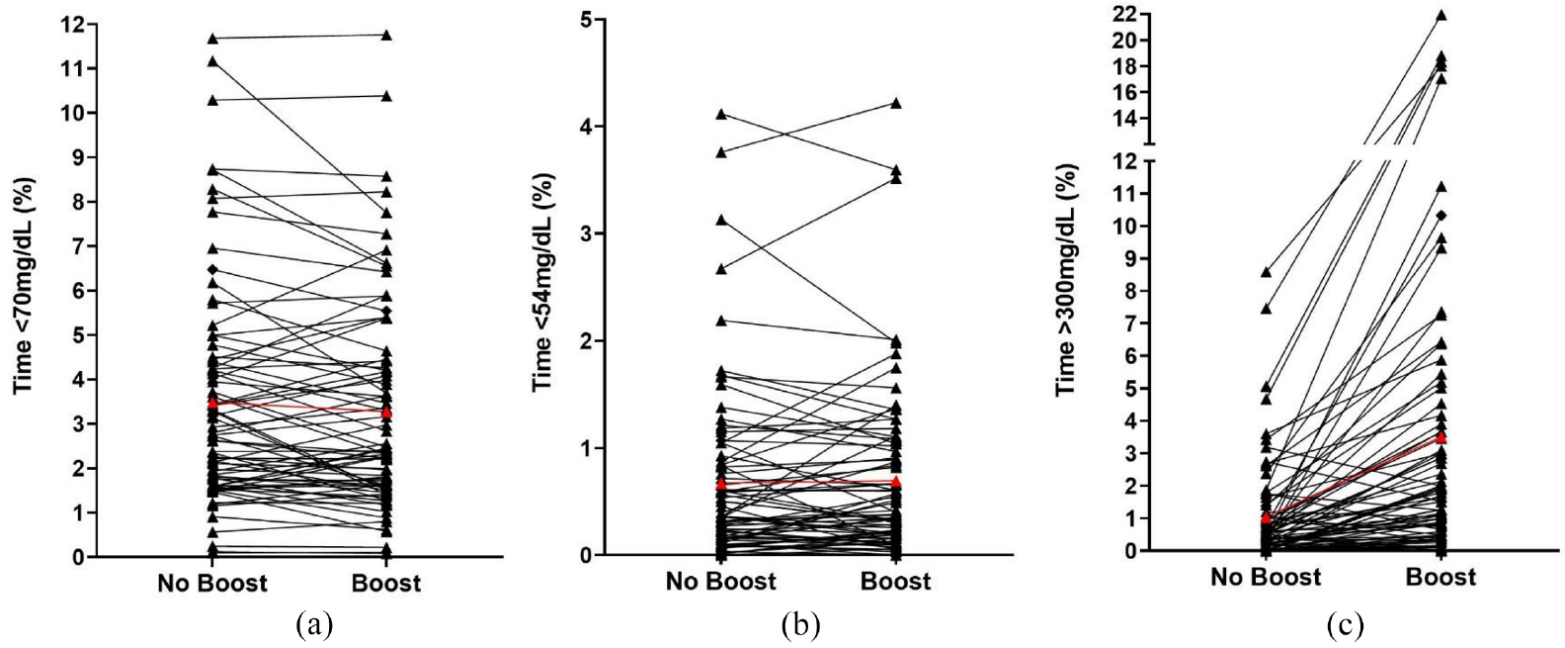
Individual participants’ time spent with glucose in level 1 hypoglycemia <70 mg/dL (a), overall mean shown in red; level 2 hypoglycemia <54 mg/dL (b), overall mean shown in red; and in significant hyperglycemia >300 mg/dL (c), overall mean shown in red, during days when no “Boost” was used and days when “Boost” was used ≥30 minutes.

There were 3933 available days for analysis, of which 1785 (45%) had ≥ 30 minutes “Boost” use (Table 2). On “Boost” days, “Boost” mode was activated for a median 103 minutes (83-151). There was no difference in time spent in hypoglycemia <70 and <54 mg/dL between “Boost” days (days with ≥30 minutes “Boost” use) and “non-Boost” days; mean difference –0.10% (95% confidence interval [CI] –0.28 to 0.07; *P* = .249) time <70 mg/dL, and 0.03 (–0.04 to 0.09; *P* = .416) time <54 mg/dL. Time in significant hyperglycemia >300 mg/dL was 1.39 percentage points (1.01-1.77; *P* < .001) higher on “Boost” days, while time in target range 70 to 180 mg/dL was 9.5 percentage points (7.2-11.8; *P* < .001) lower and mean glucose 14.6 mg/dL (12.1-16.9; *P* < .001) higher on “Boost” days. In addition, glucose variability was higher on “Boost” days with mean coefficient of variation of glucose 35%, compared with 31% on “non-Boost” days (mean difference: 3.9% [3.2-4.6, *P* < .001]). Figure 1 shows the trend of time spent in hypoglycemia <70 and <54 mg/dL and time spent in significant hyperglycemia >300 mg/dL per participant on “Boost” versus “non-Boost” days. The observed increase in time spent in significant hyperglycemia >300 mg/dL on “Boost” days was not unexpected and is further explored in the “Discussion.”

**Table 2.** Comparison of Main Glycemic Outcomes on Boost and Non-Boost Days.

	Boost days	Non-boost days	Mean difference [95% confidence interval]	*P* value
Total available days	1785	2148	—	—
Number of days available per participant	23 (15, 29)	28 (20, 37)	—	—
Time using “Boost” (%)	7.2 (5.7, 10.5)	—	—	—
Time using “Boost” (minutes)	103 (83, 151)	—	—	—
Time in hypoglycemia (%)				
<70 mg/dL	3.18 ± 1.91	3.28 ± 1.88	−0.10 [−0.28, 0.07]	.249
<54 mg/dL	0.60 ± 0.51	0.57 ± 0.51	0.03 [−0.04, 0.09]	.416
Time in significant hyperglycemia >300 mg/dL (%)	2.37 ± 2.11	0.98 ± 1.36	1.39 [1.01, 1.77]	<.001
Time in target range 70-180 mg/dL (%)	69.1 ± 13.2	78.6 ± 8.9	−9.5 [−11.8, −7.2]	<.001
Mean sensor glucose (mg/dL)	153.3 ± 19.6	138.9 ± 14.8	14.6 [12.1, 16.9]	<.001
Coefficient of variation of glucose (%)	35.2 ± 5.2	31.2 ± 4.9	3.9 [3.2, 4.6]	<.001

Data are mean ± SD or median (IQR). N = 76 participants.

Abbreviations: IQR, interquartile range; SD, standard deviation.

## Discussion

The present study reports on the safety of a unique user-initiated insulin delivery intensification mode (“Boost”) during eight weeks of home-use of the Cambridge HCL algorithm by children and adults with type 1 diabetes. There was no difference in time spent in hypoglycemia on days when “Boost” was used compared with “non-Boost” days. This suggests that user-initiated closed-loop insulin delivery intensification is safe to use across all age groups.

The addition of personalizable features in HCL systems is an evolving area. In qualitative studies exploring peoples’ experience using different closed-loop systems, users reported wanting to actively collaborate with the system, to improve glycemic control on days with atypical glucose excursions.^9,10,14^ Intrinsic day-to-day variability in insulin requirements^15,16^ can be exacerbated by a variety of factors including intercurrent illness, hormonal fluctuations, and challenging meals.^16,17^ Closed-loop systems aim to address variability by automatically adjusting insulin delivery based on real-time sensor glucose values, but are more limited in their ability to cope with extreme glucose levels, in contrast to users’ expectations.^8^ Even in the context of systems incorporating adaptive learning, users felt that being able to communicate new information to the algorithm and influence insulin delivery was important in terms of managing glucose levels at times when the system might not be “aggressive” enough on its own.^9,18^

“Boost” mode enables users to notify the closed-loop algorithm that insulin requirements are higher than usual. In our study, time spent in significant hyperglycemia >300 mg/dL was higher on “Boost” days, with lower time in target range and higher mean glucose. This was an expected result and suggests that participants use “Boost” on days with more extreme hyperglycemic excursions. An episode of significant hyperglycemia is likely the trigger for “Boost” activation, explaining the higher percentage time >300 mg/dL on days when “Boost” mode was used. In keeping with this suggestion, our results showed that there were fewer “Boost” days than “non-Boost” days, with the majority of participant days not including use of “Boost.” This is reflective of the closed-loop system’s general ability to maintain glucose in the target range at default settings without need for additional user-input, as evidenced by an overall mean time of 75% with glucose in the target range of 70 to 180 mg/dL. The suggestion that “Boost” is primarily used on days with significant hyperglycemia was corroborated by qualitative study findings, where participants reported that activating “Boost” helped the closed-loop system to manage minor illness and atypical hyperglycemia events more effectively.^10,19^ Improving the closed-loop system’s ability to cope with significant hyperglycemia events may help to improve overall glycemic control and system usability.

Incorporating user-initiated increased insulin delivery into the closed-loop algorithm itself has potential safety benefits. Using manual insulin correction doses or temporary basal rates to manage significant hyperglycemia carries an inherent risk of resultant hypoglycemia.^7^ Appropriate dosing decisions rely on the accuracy of a range of settings, as well as the timing of the corrective insulin dose itself, making dosing decisions a challenging task.^7^ In contrast “Boost” enables an increase in algorithm-driven insulin delivery adjusted continuously based on sensor glucose values, with the algorithm using parameters based on adaptive learning, rather than pre-defined settings. When glucose levels have returned to target, “Boost” becomes inactive, regardless of pre-programmed duration. In our study, there was no difference in time spent in hypoglycemia on “Boost” days, emphasizing the potential benefit of advanced technologies in terms of managing extreme glucose excursions safely.

The strengths of our study include the broad age-range of participants between 1 and 80 years and the multicenter, multinational study design with home use of the HCL system without remote monitoring. The longer eight-week study period has been shown to provide representative data for mean glucose, glucose variability, and time spent with glucose below, within, and above the target range,^20^ supporting the generalizability of our findings. We included only participants with at least 10 “Boost” days and 10 “non-Boost” days, reducing the risk of selection bias. Limitations include the retrospective analysis and the fact that time spent with “Boost” active and time spent in hypoglycemia was calculated on a day-by-day basis rather than an individual event basis. This approach does not allow for any assessment of efficacy of “Boost” and has the potential of underestimating delayed hypoglycemia events following “Boost”-use.

## Conclusions

In summary, the use of a user-initiated insulin delivery intensification mode is safe in children, adolescents, adults, and older adults with type 1 diabetes using the Cambridge HCL algorithm. Increasingly, users expect systems to be personalizable as well as individually adaptable. Further studies are warranted to assess whether such personalizable closed-loop features could improve glycemic control while maintaining the safe use of these systems.

## References

[1] Ware J Hovorka R. Recent advances in closed-loop insulin delivery. Metabolism. 2022;127:154953. 34890648 10.1016/j.metabol.2021.154953 PMC8792215

[2] Boughton CK Hovorka R. New closed-loop insulin systems. Diabetologia. 2021;64(5):1007-1015. 33550442 10.1007/s00125-021-05391-w PMC8012332

[3] Brown SA Kovatchev BP Raghinaru D , et al. Six-month randomized, multicenter trial of closed-loop control in type 1 diabetes. N Engl J Med. 2019;381(18):1707-1717. 31618560 10.1056/NEJMoa1907863 PMC7076915

[bibr4-19322968221141924] Ware J Allen JM Boughton CK , et al. Randomized trial of closed-loop control in very young children with type 1 diabetes. N Engl J Med. 2022;386(3):209-219. 35045227 10.1056/NEJMoa2111673

[bibr5-19322968221141924] Boughton CK Hartnell S Thabit H , et al. Hybrid closed-loop glucose control compared with sensor augmented pump therapy in older adults with type 1 diabetes: an open-label multicentre, multinational, randomised, crossover study. Lancet Healthy Longev. 2022;3(3):e135-e142. 35359882 10.1016/S2666-7568(22)00005-8 PMC8967297

[6] Abraham MB de Bock M Smith GJ , et al. Effect of a hybrid closed-loop system on glycemic and psychosocial outcomes in children and adolescents with type 1 diabetes: a randomized clinical trial. JAMA Pediatr. 2021;175(12):1227-1235. 34633418 10.1001/jamapediatrics.2021.3965 PMC8506294

[7] Cryer PE Davis SN Shamoon H. Hypoglycemia in diabetes. Diabetes Care. 2003;26(6):1902-1912. 12766131 10.2337/diacare.26.6.1902

[8] Hendrieckx C Poole LA Sharifi A , et al. “It is definitely a game changer”: a qualitative study of experiences with in-home overnight closed-loop technology among adults with type 1 diabetes. Diabetes Technol Ther. 2017;19(7):410-416. 28537437 10.1089/dia.2017.0007

[bibr9-19322968221141924] Lawton J Blackburn M Rankin D , et al. Participants’ experiences of, and views about, daytime use of a day-and-night hybrid closed-loop system in real life settings: longitudinal qualitative study. Diabetes Technol Ther. 2019;21(3):119-127. 30720338 10.1089/dia.2018.0306 PMC6434584

[10] Kimbell B Rankin D Hart RI , et al. Parents’ experiences of using a hybrid closed-loop system (CamAPS FX) to care for a very young child with type 1 diabetes: qualitative study. Diabetes Res Clin Pract. 2022;187:109877. 35469973 10.1016/j.diabres.2022.109877

[11] Leelarathna L Choudhary P Wilmot EG , et al. Hybrid closed-loop therapy: where are we in 2021? Diabetes Obes Metab. 2021;23:655-660. 33269551 10.1111/dom.14273

[12] Boughton CK Hartnell S Thabit H , et al. Hybrid closed-loop glucose control with faster insulin aspart compared with standard insulin aspart in adults with type 1 diabetes: a double-blind, multicentre, multinational, randomized, crossover study. Diabetes Obes Metab. 2021;23(6):1389-1396. 33606901 10.1111/dom.14355 PMC11497277

[13] Boughton CK Allen JM Ware J , et al. Closed-loop therapy and preservation of C-peptide secretion in type 1 diabetes. N Engl J Med. 2022;387(10):882-893. 36069870 10.1056/NEJMoa2203496

[14] Musolino G Dovc K Boughton CK , et al. Reduced burden of diabetes and improved quality of life: experiences from unrestricted day-and-night hybrid closed-loop use in very young children with type 1 diabetes. Pediatr Diabetes. 2019;20(6):794-799. 31140654 10.1111/pedi.12872 PMC6771658

[15] Dovc K Boughton C Tauschmann M , et al. Young children have higher variability of insulin requirements: observations during hybrid closed-loop insulin delivery. Diabetes Care. 2019;42(7):1344-1347. 31221700 10.2337/dc18-2625 PMC6609966

[16] Ruan Y Thabit H Leelarathna L , et al. Variability of insulin requirements over 12 weeks of closed-loop insulin delivery in adults with type 1 diabetes. Diabetes Care. 2016;39(5):830-832. 26965717 10.2337/dc15-2623

[17] Wolpert HA Atakov-Castillo A Smith SA Steil GM. Dietary fat acutely increases glucose concentrations and insulin requirements in patients with type 1 diabetes: implications for carbohydrate-based bolus dose calculation and intensive diabetes management. Diabetes Care. 2013;36(4):810-816. 23193216 10.2337/dc12-0092 PMC3609492

[18] Iturralde E Tanenbaum ML Hanes SJ , et al. Expectations and attitudes of individuals with type 1 diabetes after using a hybrid closed loop system. Diabetes Educ. 2017;43(2):223-232. 28340542 10.1177/0145721717697244 PMC7162535

[19] Rankin D. Adolescents’ experiences of using a smartphone application hosting a closed-loop algorithm to manage type 1 diabetes in everyday life: qualitative study. J Diabetes Sci Technol. 2021;15:1042-1051. 34261348 10.1177/1932296821994201 PMC8411472

[20] Leelarathna L Thabit H Willinska ME , et al. Duration of hybrid closed-loop insulin therapy to achieve representative glycemic outcomes in adults with type 1 diabetes. Diabetes Care. 2020;43(3):e38-e39. 31949086 10.2337/dc19-2041

